# Migraine and white matter lesions: a mendelian randomization study

**DOI:** 10.1038/s41598-023-38182-x

**Published:** 2023-07-06

**Authors:** Junyan Huo, Gan Zhang, Wenjing Wang, Wen Cao, Mengxia Wan, Tao Huang, Dongsheng Fan, Yu Fu

**Affiliations:** 1 Department of Neurology, Peking University Third Hospital, No. 49, North Garden Rd., Haidian District, Beijing, 100191 China; 2 Department of Epidemiology and Biostatistics, School of Public Health, Peking University, Beijing, China; 3 Key Laboratory of Molecular Cardiovascular Sciences, Peking University, Ministry of Education, Beijing, China

**Keywords:** Diseases, Neurology

## Abstract

Previous studies have found that migraine patients are associated with white matter lesions (WMLs), but the causal relationship between the two remains unclear. We intend to explore the bidirectional causal relationship between migraine and WMLs using a two-sample mendelian randomization (MR) method. We employed summary-level data from a recent large-scale genome-wide association study (GWAS) that characterized three white matter (WM) phenotypes: white matter hyperintensities (WMH, N = 18,381), fractional anisotropy (FA, N = 17,673), and mean diffusivity (MD, N = 17,467), as well as migraine (N = 589,356). The inverse variance-weighted (IVW) method was used as the main approach for analyzing causality. Weighted median analysis, simple median analysis, and MR-Egger regression served as complementary methods. The bidirectional MR study affords no support for causality between WMLs and migraine. In all MR methods, there was no obvious causal evidence between them. In our bidirectional MR study, we didn't reach this conclusion that WMLs can cause migraine, migraine wouldn’t increase the risk of WMLs, either.

## Introduction

Migraine is one of the most common complaints in the outpatient department. The World Health Organization ranks migraine as the third most common and second most disabling neurological disorder in the world^1,2^. Migraine is a chronic disease manifested as recurrent moderate to severe headaches^3^, migraine attack is often accompanied by nausea, vomiting, photophobia and fear of sound, and its symptoms generally last for 4–72 h^4^, which deteriorated patients’ quality of life. The routine examination of migraine patients often has no positive findings, but with the popularization of magnetic resonance imaging (MRI), more and more migraineurs have found WMLs, These WMHs can be seen on T2 weighted or fluid attenuated inversion recovery (FLAIR) sequences and are usually distributed in the deep or periventricular white matter, showing multiple, small, punctate lesions. Adult migraineurs have two to four times the risk of concurrent white matter lesions compared to those without migraine^5^. In some cases, WMLs can be diagnosed a potential disease, and migraine may be just a symptom^6^. Although WMLs are often found in patients with migraine, population-based studies show that migraine may be a potential risk factor for WMLs^7,8^, but not all migraineurs present with WMLs on MRI, the potential mechanisms remain unclear^9^. Despite the prevalence of migraine-associated white matter lesions, their pathophysiology remains poorly understood^10^, The relationship between the two has always troubled us and is highly worthy of further research. The evidence is primarily based on observational research^11–13^, but the direct clues to the causal relationship between them are still uncertain. There is a need to employ a suitable method to explore whether migraine plays a key role in white matter lesions, and vice versa.

MR is an approach of using genetic variation to estimate the causal association between exposure and outcome^14^. It relies on the natural, random assignment of genetic variants during meiosis, producing a random distribution of genetic variants in the population. Individuals inherit genetic variants affecting or not affecting risk factors at birth and are then recorded as having the disease or not, and since these genetic variants are usually not associated with confounding factors, differences in outcomes between carried and uncarried variants can be attributed to differences in risk factors^15^. The principle of MR is based on Mendelian genetic law and instrumental variables (IVs) estimation method. The mendelian randomization method uses genetic variation as IVs to derive the causal relationship between outcomes and exposures, which can effectively avoid the confounding bias of traditional epidemiological studies^16^. Thus, we used a two-sample MR design to assess whether migraine increases white matter lesions and to explore whether WMLs increase the risk of migraine.

## Materials and methods

### GWAS summary statistics for WMLs

Summary-level GWAS data of MRI characteristics of white matter were acquired from the UK Biobank (UKB)^17^. They are all of European ancestry, recruited at the age of 40–69 years and the time span is from 2016 to October 2018^18^. The image data covers approximately 20,000 individuals, the summary-level GWAS data was publicly available and accurate individual image information could be applied to the organization. This GWAS consisted of the next three MRI markers: white matter hyperintensities data acquired in 18,381 Europeans, fractional anisotropy data acquired in 17,673 Europeans and mean diffusivity data obtained from 17,467 Europeans. WMH traits were log-transformed and normalized to brain volume, the reduced white matter integrity may not be observed by conventional MRI, but can be imaged with alternative imaging techniques, such as diffusion tensor imaging (DTI)^19^. FA mainly responds to the integrity of fiber bundles, and MD mainly represents damage to axons and myelin sheaths. A decrease in FA values and an increase in MD values indicate that the microstructure of white matter is disrupted^20^. To obtain a single measurement of FA and MD for the entire white matter from DTI images, FactoMineR was used to perform principal component analysis (PCA) of FA and MD measurements for different brain bundles analyzed^17^. Among these data, people with central nervous system (CNS) disorders that specifically cause white matter lesions were excluded from the downstream association analyses, such as stoke, Parkinson’s disease, multiple sclerosis, dementia, or any other degenerative disease of CNS were excluded. We can obtain summary-level GWAS data for white matter MRI characteristics from the Cerebrovascular Disease Knowledge Portal (https://www.cerebrovascularportal.org).

### GWAS summary statistics for migraine

We acquired GWAS summary-level data for migraine from the International Headache Genetics Consortium (IHGC), which included 48,975 cases and 540,381 controls of European ancestry, except for 23andMe^21^. This is the largest GWAS data of migraine so far. Two methods were used to identify migraine cases in the entire cohort, including self-report and compliance with ICHD-II criteria. Self-reported migraine was categorized based on responses to the initial 1998 questionnaire, “Has a doctor ever said that you suffer or have suffered from ……” (Yes/No)^22^.

### Selection of migraine and white matter lesions IVs

Single nucleotide polymorphisms (SNPs) that reached genome-wide significant levels (*p* < 5 × 10^−8^) were selected from the exposed GWAS, and to avoid bias caused by relevant instrumental variables, we removed SNPs with linkage disequilibrium based on the r^2^ < 0.001 linkage disequilibrium threshold within 1000 kb as instrumental variables for the final MR study. We used the F statistic of each SNP (F = beta^2^/se^2^) to assess the power for SNPs, and SNPs with lower statistical power were removed^23^. SNPs that were not available for outcome data were replaced by proxies (r^2^ > 0.9), we can get the proxies by searching the website SNIPA (https://snipa.helmholtzmuenchen.de/snipa3/), each trait that we selected of IVs was shown in supplemental Tables 1 and 2.

## Mendelian randomization analysis

**Figure 1:**
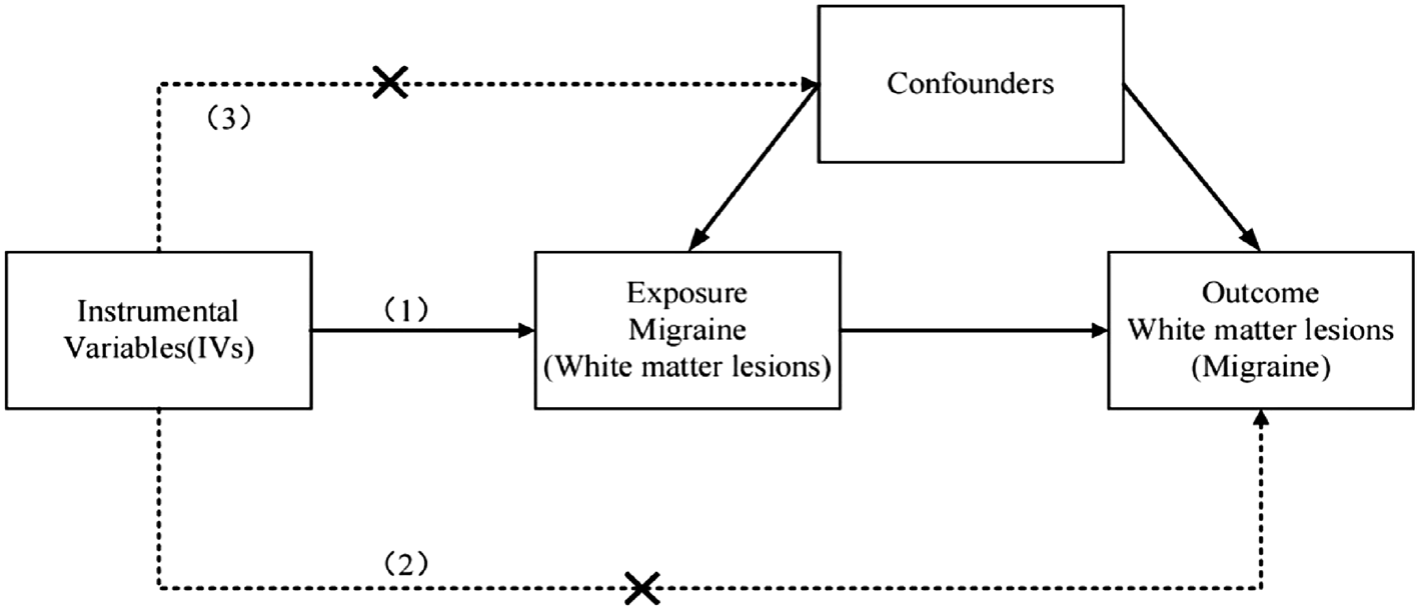
Design and main assumptions of our Mendelian randomization study.

The MR should be carried out under three basic assumptions: (1) The genetic variation is strongly associated with exposure; (2) the genetic variation is independent of outcome except by means of exposure; and (3) genetic variation is independent of any potential confounding factors^16^ (Fig. 1). For each direction of potential effect, the main MR estimates were performed using IVW method^24^. MR-Egger, weighted-median methods and simple-median methods were used as supplementary methods to IVW. The MR-Egger intercept was used to detect horizontal pleiotropy^25^, we also used Pleiotropy Residual Sum and Outlier (MR-PRESSO) to assess horizontal pleiotropic outliers^26^. The Cochrane’s Q value was used to evaluate the heterogeneity. All of our analyses were conducted by the ‘‘Two sample MR’’ and ‘‘MR-PRESSO’’ packages in version R 4.0.3. Results are expressed as odds ratios (ORs) per an approximate 1 standard deviation (SD) raise of exposure. *P* < 0.05 was considered as statistical significance.

## Results

### Effect of WMLs traits on migraine and sensitivity analyses of WMLs traits on migraine

**Figure 2:**
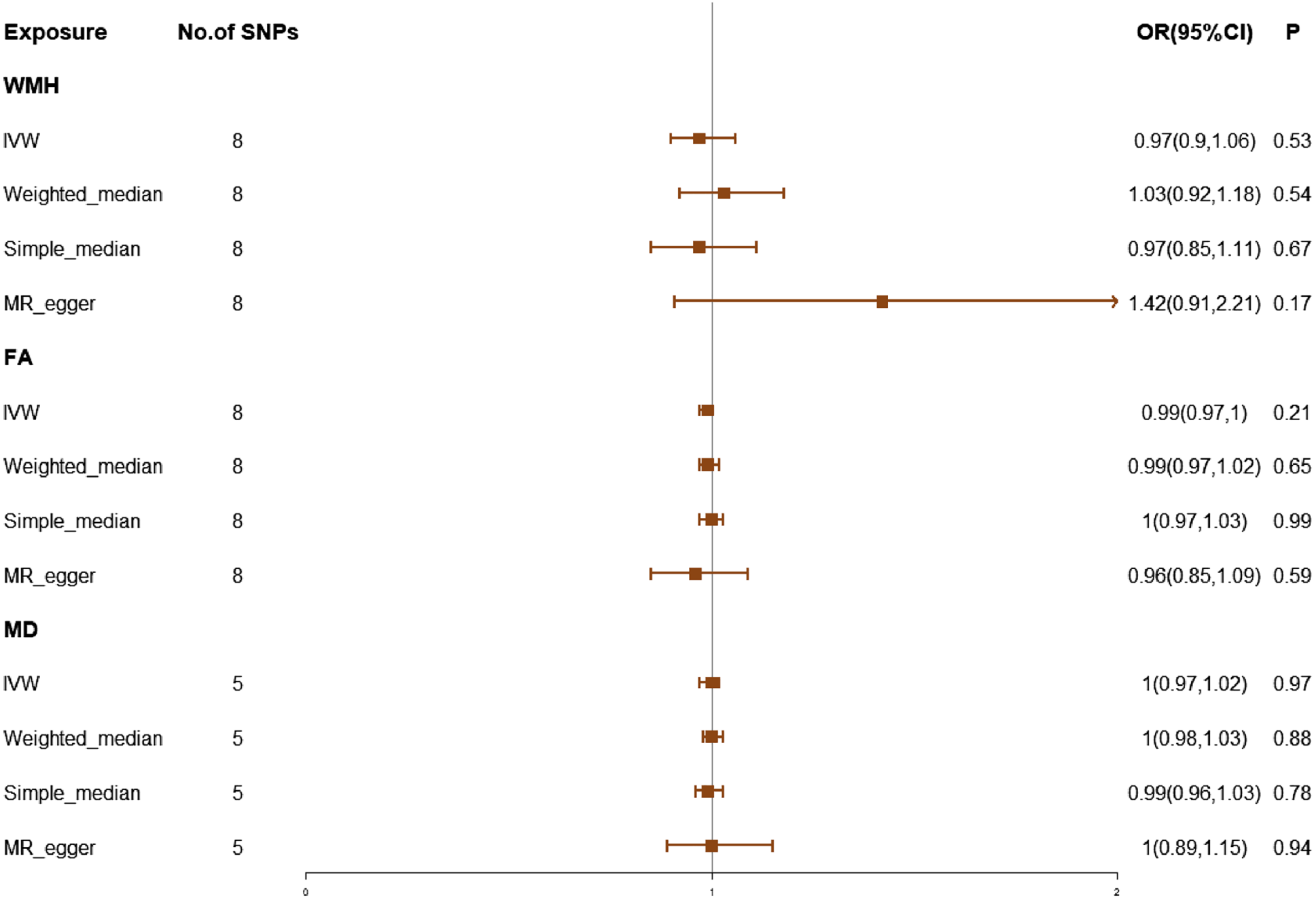
The causal effects of white matter lesions on migraine using two-sample Mendelian randomization methods. IVW, inverse variance weighted; SNPs, single nucleotide polymorphisms; OR, odds ratios; CI, confidence interval; WMH, white matter hyperintensities; FA, fractioal anisotropy; MD, mean diffusivity.

We identified 8,5 and 8 SNPs as IVs in our MR analysis of migraine on WMLs phenotypes FA, MD, and WMH, respectively. The results were as follows: FA (OR, 0.99; 95% CI 0.97–1; *P* = 0.21), MD (OR, 1; 95% CI 0.97–1.02; *P* = 0.97), or WMH (OR, 0.97; 95% CI 0.9–1.06; *P* = 0.53), (Fig. 2), we didn’t find any effect of WMLs on migraine. Supplementary Table 1 and 2 provide an expression of IVs of each exposure trait (including number of independent SNPs, F-statistic) and demonstrate a description of the summary statistics for the genetic variants associated with migraine and WMLs.

In the direction of MR analysis of white matter lesions on migraine, for FA, MD and WMH as exposures, the p value of Cochran’s Q was < 0.05 (Supplementary Table 4, However, no causal effect was found between WMH and migraine outcomes even when heterogeneity was taken into account using a random effects model. The method of sensitivity analysis showed no horizontal pleiotropy.

### Effect of migraine on WMLs and sensitivity analyses of migraine on WMLs

**Figure 3:**
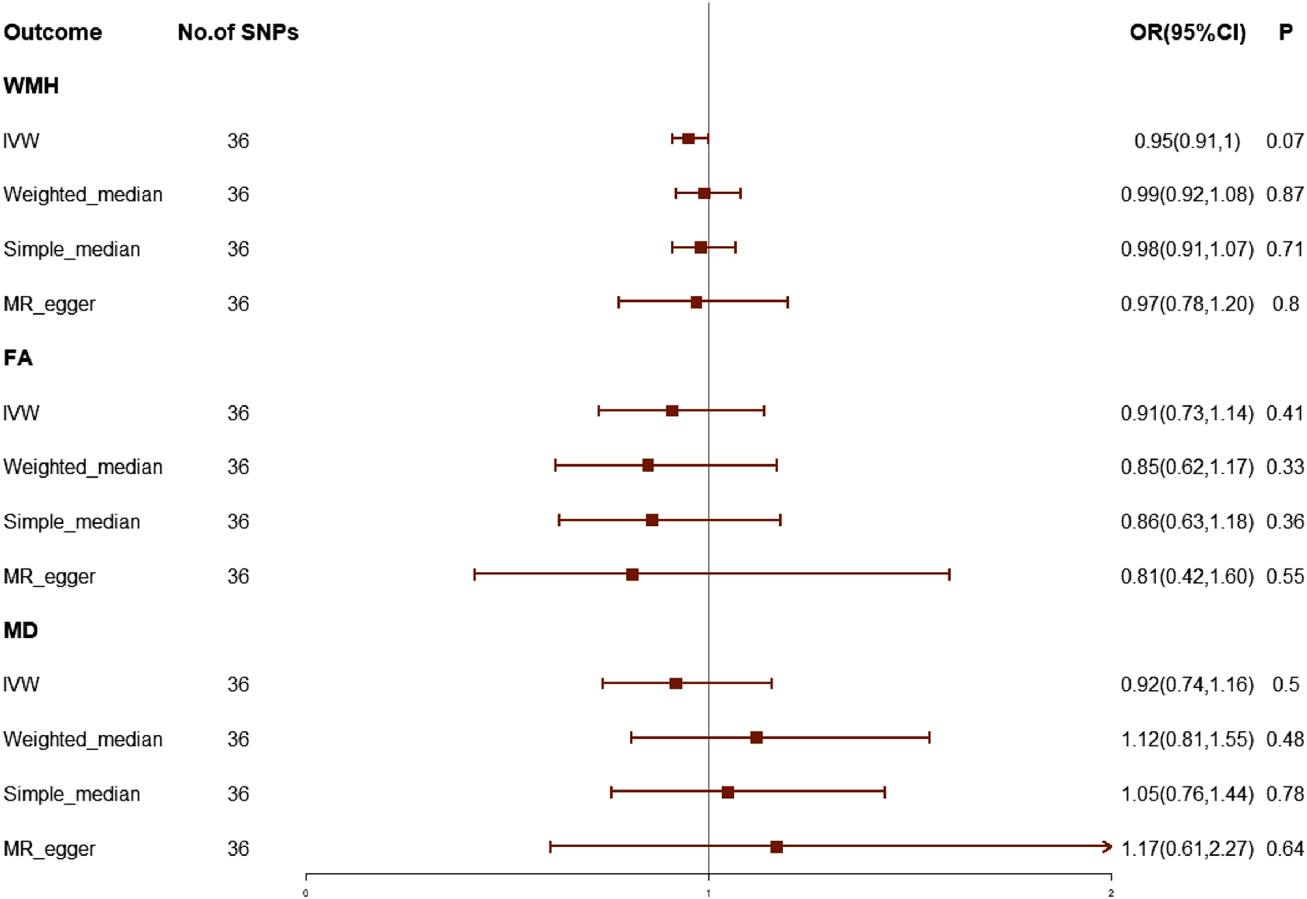
The causal effects of migraine on white matter lesions using two-sample mendelian randomization methods. IVW, inverse variance weighted; SNPs, single nucleotide polymorphisms; OR, odds ratios; CI, confidence interval. WMH, white matter hyperintensities; FA, fractioal anisotropy; MD, mean diffusivity.

According to the criteria of MR design, we finally selected 36 SNPs as IVs in the MR analysis of migraine on white matter lesions (Supplementary Table 1). We found no association of migraine on white matter lesions applying the inverse-variance-weighted method, the IVW results for the three WMLs phenotypes were as follows: migraine on FA (OR 0.91; 95% CI 0.73–1.14; *P* = 0.41), migraine on MD (OR 0.92; 95% CI 0.74–1.16; *P* = 0.5), or migraine versus WMH (OR 0.95; 95% CI 0.91–1; *P* = 0.07) (Fig. 3).

In the MR analysis of migraine versus white matter lesion orientation, for FA and MD as outcomes, the p values of Cochran’s Q was 0.38 and 0.52, indicating no heterogeneity in this MR analysis, and for WMH as outcomes, the P values of Cochran’s Q was < 0.05, which indicates the presence of heterogeneity (Supplementary Table 3), and when the heterogeneity was taken into account, we used the multiplicative random-effects inverse-variance-weighted methods as a supplementary method, but still no causal relationship was found. The MR-Egger intercept and The MR PRESSO test together suggest no evidence of possible pleiotropic effects.

We conducted a lot of sensitivity analyses to assess the robustness and consistency of the results. In different sensitivity analyses, we obtained consistent estimates of the impact of migraine on white matter lesions and vice versa. Instrumental variables on the risk of white matter lesions and migraine showed the consistent and directional effects. In addition, the results of the leave-one-out analysis showed that the whole estimates were not driven by single SNP, but the overall combined effect between migraine and white matter lesions (Supplementary Fig. 1–18).

## Discussion

The relationship between white matter lesions and migraine has been controversial for several years. We used a comprehensive MR analysis to explore the relationship between them. Both had the largest GWAS sample sizes to date. Unfortunately, we did not observe that WMLs could increase the risk of migraine. Similarly, genetically predicted migraine did not have a causal effect on white matter lesions. As far as we know, it is the first MR study about the relationship between white matter lesion and migraine.

WMLs is an imaging academic term, which was first proposed by Professor Hachinski in 1987. It is used to describe the low-density shadow of brain white matter found by computed tomography or MRI T2 weighted and FLAIR sequences, often showing high signal in the periventricular and subcortical areas^27^, the pathogenesis of white matter lesions is still unclear, and some studies suggest that it is related to age, hypertension, immune response, and neuroinflammation^28^. Previous observational studies have reported an association between white matter lesions and migraine. Swartz and Kern et al. published a meta-analysis based on the results of seven case–control studies. The results showed that patients with migraine had a higher risk of white matter damage than those without migraine^29^. The burden of white matter lesions in migraine is 2–4 times higher than that of the general population^10^. An Epidemiological study of brain abnormalities, with a primary study population of Danes aged 30–60 years, showed an increased risk of deep white matter lesions in female migraineurs^30^, a study consisted of 780 elderly participants with a mean age of 69 years. It showed that migraine with aura was associated with an accumulation of white matter lesions^31^. These studies suggest that migraine can increase the risk of white matter lesions. However, they are mainly cohort and case–control studies, and do not explain the causal relationship between the two, and the level of evidence between them is insufficient. Furthermore, both migraine and cerebral white matter lesions are diseases of complex etiology, and the pathogenesis of both is currently unclear, and conducting cross-sectional studies may introduces interference from multiple confounding factors. Lastly, some studies are inconsistent in their classification of white matter lesions.

In this study, we assessed the causal association between migraine and white matter lesions using MR method. We find no causal relationship between them, which can be explained in several ways. First, white matter lesions and migraine may be comorbid, and both share common risk factors, such as smoking. Second, White matter changes appeared to be benign and nonprogressive in individuals with migraine. A longitudinal MRI study over 8–12 years showed no association between migraine and WMH progression over time^12^. Our results are consistent with a prospective study that WMLs did not differ from controls in children with or without migraine aura^32^. Third, previous study shows that migraine with aura may be a risk factor for WMLs, and although our study did not carry out classification analysis, it included migraine with aura. A study with a population-based sample of female twins aged 30–60 years in Denmark showed no association between WML and migraine aura^33^, which is consistent with our results. Last, Many migraineurs have a combination of patent foramen ovale (PFO), which may be an intermediate mediator that leads to white matter lesions in the brain^34^. Further studies on the pathophysiology of migraine and white matter lesions are warranted.

One of the advantages of our research is the use of a Mendelian randomization design, by using randomly assigned genetic variations as IVs, our research has greatly reduced confusion or reversed causal bias, so it produced convincing results. Furthermore, because the analysis was limited to people of European ancestry, biases brought by demographic structure are impossible to influence our results. Other advantages include the consistency of causal estimates using multiple analytical methods, and the robust estimation effect of each IVs (all F-statistics > 10). In addition, The GWAS data we use has a large sample size, the study was reported according to the statement of STROBE-MR.

Our study has several limitations. First, self-reported migraine is not as reliable as the objective scale or physician's recent diagnosis and may have some bias, including patients with a larger sample size who meet the diagnostic criteria for migraine can reduce bias. Second, because the subject in this study was limited to Europe, this conclusion may not apply to non-European people. Studies with larger sample sizes of multi-ethnic GWAS data are needed for further extrapolation to humans. Finally, the three key assumptions of MR study are relatively strict and difficult to meet in practice, both migraine and white matter lesions both have complex etiologies. It may not be possible to measure all exposure-related confounders and to completely eliminate biased estimates of causal inference.

## Conclusions

In conclusion, this study does not find a causal relationship between migraine and white matter lesions. Further efforts are still needed to investigate the pathophysiology between migraine and white matter lesions.

## Supplementary Information

Supplementary Information 1.

Supplementary Information 2.

## Data Availability

The GWAS data of migraine used during the current study can be obtained from cited study authors on reasonable request and the GWAS data of white matter lesions are available on the Cerebrovascular Disease Knowledge Portal: https://www.cerebrovascularportal.org.
