# Supplementary material for: Migraine and white matter lesions: a mendelian randomization study
Source: Sci Rep. 2023 Jul 6;13:10984. doi: 10.1038/s41598-023-38182-x (PMC10326014; doi:10.1038/s41598-023-38182-x)

**Supplementary materials**

Supplement table 1: Summarized data of the migraine-associated SNPs finally identified as IVs in our MR analyses.

Supplement table 2: Summarized data of the WMLs-associated SNPs finally identified as IVs in our MR analyses.

Supplement table 3: Results for MR-Egger (intercept), MR-PRESSO and Cochran's Q test of migraine for WMLs.

Supplement table 4: Results for MR-Egger (intercept), MR-PRESSO and Cochran's Q test of WMLs for migraine

Supplement figure 1: Scatter plots of the causal effects of migraine with risk for FA

Supplement figure 2: Scatter plots of the causal effects of migraine with risk for MD

Supplement figure 3: Scatter plots of the causal effects of migraine with risk for WMH

Supplement figure 4: Forest plots of the causal effects between migraine-related SNPs and FA

Supplement figure 5: Forest plots of the causal effects between migraine-related SNPs and MD

Supplement figure 6: Forest plots of the causal effects between migraine-related SNPs and WMH

Supplement figure 7: Leave-one-out sensitivity analysis for migraine on FA

Supplement figure 8: Leave-one-out sensitivity analysis for migraine on MD

Supplement figure 9: Leave-one-out sensitivity analysis for migraine on WMH

Supplement figure10: Scatter plots of the causal effects of FA with risk for migraine

Supplement figure11: Scatter plots of the causal effects of MD with risk for migraine

Supplement figure12: Scatter plots of the causal effects of WMH with risk for migraine

Supplement figure 13: Forest plots of the causal effects between FA SNPs and migraine

Supplement figure 14: Forest plots of the causal effects between MD SNPs and migraine

Supplement figure 15: Forest plots of the causal effects between WMH SNPs and migraine

Supplement figure16: Leave-one-out sensitivity analysis for FA on migraine

Supplement figure17: Leave-one-out sensitivity analysis for MD on migraine

Supplement figure18: Leave-one-out sensitivity analysis for WMH on migraine

Supplement table 1. Summarized data of the migraine-associated SNPs finally identified as IVs in our MR analyses

| **SNP** | **Chr** | **Pos** | **Effect Allele** | **Other Allele** | **EAF** | **Beta Estimate** | **SE** | **F-statistic** | **P value** | **beta.WMLs** | **se.WMLs** | **eaf.WMLs** | **Pval.WMLs** |
| --- | --- | --- | --- | --- | --- | --- | --- | --- | --- | --- | --- | --- | --- |
| rs10166942 | 2 | 2.35E+08 | C | T | 0.19655 | -0.11471 | 0.00974 | 138.59359 | 5.89E-32 | -0.00572 | 0.01222 | 0.19281 | 0.63970 |
| rs1019990 | 18 | 4.49E+07 | T | C | 0.29857 | -0.05541 | 0.00838 | 43.74801 | 3.87E-11 | 0.01208 | 0.01051 | 0.29916 | 0.25032 |
| rs10234636 | 7 | 4.04E+07 | T | C | 0.11364 | 0.10962 | 0.01168 | 88.10167 | 6.60E-21 | -0.00819 | 0.01565 | 0.10700 | 0.60050 |
| rs1025497 | 19 | 4.19E+07 | A | G | 0.37343 | -0.04993 | 0.00791 | 39.81127 | 2.89E-10 | -0.01959 | 0.00992 | 0.37215 | 0.04825 |
| rs10456100 | 6 | 3.92E+07 | T | C | 0.28291 | 0.06073 | 0.00847 | 51.42798 | 7.75E-13 | -0.01598 | 0.01072 | 0.28176 | 0.13597 |
| rs10833535 | 11 | 3.26E+06 | A | G | 0.47566 | 0.05296 | 0.00771 | 47.13426 | 6.89E-12 | -0.01257 | 0.00965 | 0.46690 | 0.19265 |
| rs10849061 | 12 | 4.52E+06 | C | T | 0.48628 | 0.06316 | 0.00759 | 69.20390 | 9.36E-17 | 0.01529 | 0.00965 | 0.48917 | 0.11292 |
| rs11153082 | 6 | 9.71E+07 | G | A | 0.32358 | 0.09770 | 0.00804 | 147.70458 | 6.02E-34 | -0.04635 | 0.01040 | 0.31603 | 0.00001 |
| rs11172113 | 12 | 5.75E+07 | C | T | 0.42260 | -0.11737 | 0.00776 | 228.92700 | 1.15E-51 | -0.00155 | 0.00979 | 0.41181 | 0.87402 |
| rs112255710 | 10 | 1.01E+08 | T | C | 0.07774 | -0.09936 | 0.01463 | 46.15820 | 1.13E-11 | -0.01780 | 0.01810 | 0.07907 | 0.32553 |
| rs11624776 | 14 | 9.36E+07 | C | A | 0.31320 | -0.05158 | 0.00840 | 37.74138 | 8.34E-10 | 0.00233 | 0.01027 | 0.31884 | 0.82083 |
| rs11657101 | 17 | 6.06E+07 | A | G | 0.37115 | 0.06104 | 0.00926 | 43.43808 | 4.54E-11 | -0.01989 | 0.01010 | 0.35764 | 0.04881 |
| rs11782673 | 8 | 2.73E+07 | G | A | 0.16402 | -0.05701 | 0.01033 | 30.47891 | 3.46E-08 | -0.00557 | 0.01305 | 0.16225 | 0.66924 |
| rs12025158 | 1 | 1.55E+07 | A | G | 0.35134 | 0.05088 | 0.00796 | 40.86420 | 1.69E-10 | -0.01290 | 0.00998 | 0.37010 | 0.19606 |
| rs12260436 | 10 | 1.05E+08 | C | A | 0.25035 | 0.05351 | 0.00866 | 38.20774 | 6.55E-10 | -0.00087 | 0.01119 | 0.24937 | 0.93781 |
| rs13078967 | 3 | 1.54E+08 | C | A | 0.02664 | -0.16296 | 0.02524 | 41.68864 | 1.11E-10 | 0.00040 | 0.03209 | 0.02361 | 0.99011 |
| rs17303101 | 9 | 1.19E+08 | A | G | 0.29218 | 0.06909 | 0.00842 | 67.36747 | 2.37E-16 | 0.01203 | 0.01061 | 0.29138 | 0.25713 |
| rs1925950 | 1 | 1.56E+08 | G | A | 0.35298 | 0.07604 | 0.00792 | 92.16097 | 8.49E-22 | -0.00049 | 0.01009 | 0.34651 | 0.96148 |
| rs2078371 | 1 | 1.16E+08 | C | T | 0.11813 | 0.13054 | 0.01147 | 129.64400 | 5.34E-30 | 0.00638 | 0.01504 | 0.11796 | 0.67154 |
| rs2274224 | 10 | 9.60E+07 | C | G | 0.43238 | -0.06591 | 0.00771 | 73.03023 | 1.34E-17 | 0.00023 | 0.00971 | 0.43722 | 0.98148 |
| rs2672592 | 10 | 1.24E+08 | T | G | 0.36212 | 0.04414 | 0.00786 | 31.56995 | 1.97E-08 | -0.02387 | 0.01002 | 0.36107 | 0.01722 |
| rs28451064 | 21 | 3.56E+07 | A | G | 0.13337 | -0.06596 | 0.01179 | 31.30606 | 2.26E-08 | -0.01658 | 0.01432 | 0.13470 | 0.24678 |
| rs34273564 | 6 | 7.23E+07 | T | C | 0.48278 | 0.04229 | 0.00763 | 30.71521 | 3.06E-08 | 0.01495 | 0.00971 | 0.47747 | 0.12363 |
| rs42854 | 5 | 7.50E+07 | G | C | 0.31188 | 0.06446 | 0.00809 | 63.56931 | 1.63E-15 | 0.00225 | 0.01040 | 0.30881 | 0.82873 |
| rs4910165 | 11 | 1.07E+07 | C | G | 0.32252 | -0.06638 | 0.00816 | 66.22771 | 4.23E-16 | -0.02147 | 0.01033 | 0.31639 | 0.03760 |
| rs6046147 | 20 | 1.95E+07 | T | C | 0.24889 | 0.06272 | 0.00869 | 52.06121 | 5.61E-13 | -0.01318 | 0.01104 | 0.25661 | 0.23261 |
| rs6057599 | 20 | 3.12E+07 | T | C | 0.33926 | 0.04474 | 0.00808 | 30.62809 | 3.20E-08 | -0.00145 | 0.01041 | 0.31780 | 0.88944 |
| rs6904682 | 6 | 2.21E+07 | T | C | 0.44556 | -0.04514 | 0.00764 | 34.93030 | 3.52E-09 | 0.00030 | 0.00972 | 0.43712 | 0.97574 |
| rs72926788 | 2 | 2.04E+08 | C | T | 0.03704 | -0.13026 | 0.02320 | 31.52242 | 2.02E-08 | -0.03939 | 0.02467 | 0.03931 | 0.11034 |
| rs7518255 | 1 | 3.09E+06 | A | G | 0.21801 | 0.12165 | 0.00901 | 182.30694 | 1.66E-41 | -0.01810 | 0.01153 | 0.22423 | 0.11658 |
| rs7640543 | 3 | 3.05E+07 | A | G | 0.32096 | 0.04610 | 0.00811 | 32.32627 | 1.34E-08 | -0.00933 | 0.01027 | 0.32410 | 0.36390 |
| rs7684253 | 4 | 5.77E+07 | C | T | 0.44894 | -0.04389 | 0.00765 | 32.88430 | 1.00E-08 | 0.02009 | 0.00972 | 0.44930 | 0.03874 |
| rs7757975 | 6 | 1.22E+08 | T | G | 0.15762 | 0.08949 | 0.01037 | 74.47673 | 6.47E-18 | -0.01721 | 0.01344 | 0.15326 | 0.20023 |
| rs8075138 | 17 | 4.75E+07 | T | C | 0.39747 | 0.04789 | 0.00781 | 37.56954 | 9.07E-10 | 0.02181 | 0.00993 | 0.39210 | 0.02811 |
| rs9349379 | 6 | 1.29E+07 | G | A | 0.41094 | -0.08375 | 0.00795 | 110.89920 | 6.69E-26 | 0.00126 | 0.00976 | 0.40825 | 0.89687 |
| rs950570 | 3 | 8.03E+07 | T | C | 0.07006 | 0.08473 | 0.01489 | 32.39084 | 1.29E-08 | -0.01971 | 0.01759 | 0.08144 | 0.26242 |
| rs953588 | 9 | 7.17E+07 | T | C | 0.37612 | 0.05349 | 0.00780 | 46.99856 | 7.38E-12 | -0.01803 | 0.00994 | 0.37114 | 0.06969 |

Abbreviations: Chr, chromosome; SE, standard error; Pos, position; EAF, effect allele frequency; SNP, single nucleotide polymorphism

Supplement table 2. Summarized data of the WMLs-associated SNPs finally identified as IVs in our MR analyses

| **Traits** | **SNP** | **Chr** | **Pos** | **Effect Allele** | **Other Allele** | **EAF** | **Beta Estimate** | **SE** | **F-statistic** | **P.vale** | **beta.migraine** | **se.migraine** | **eaf.migraine** | **pval.migraine** |
| --- | --- | --- | --- | --- | --- | --- | --- | --- | --- | --- | --- | --- | --- | --- |
| **FA** | rs145648066 | 6 | 2.92E+07 | C | T | 0.2424 | 0.2948 | 0.0489 | 36.3638 | 1.67E-09 | -0.00132 | 0.01117 | 0.22288 | 0.90574 |
| **FA** | rs33599 | 5 | 8.28E+07 | A | G | 0.2632 | 0.2843 | 0.0480 | 35.0678 | 3.24E-09 | 0.00808 | 0.00844 | 0.27238 | 0.33841 |
| **FA** | rs4150221 | 5 | 1.40E+08 | C | T | 0.2629 | 0.2883 | 0.0476 | 36.7189 | 1.39E-09 | 0.00136 | 0.00854 | 0.26595 | 0.87396 |
| **FA** | rs56874662 | 6 | 2.70E+07 | A | C | 0.1466 | 0.3295 | 0.0593 | 30.8820 | 2.78E-08 | 0.00848 | 0.01143 | 0.12830 | 0.45816 |
| **FA** | rs6062264 | 20 | 6.12E+07 | T | C | 0.2806 | -0.2712 | 0.0473 | 32.8427 | 1.02E-08 | -0.00366 | 0.00980 | 0.29282 | 0.70905 |
| **FA** | rs67827860 | 5 | 8.29E+07 | T | C | 0.1919 | -0.5516 | 0.0535 | 106.2585 | 7.61E-25 | 0.00522 | 0.00949 | 0.19765 | 0.58251 |
| **FA** | rs7590302 | 2 | 2.17E+08 | C | T | 0.3051 | 0.2511 | 0.0455 | 30.4612 | 3.45E-08 | -0.00575 | 0.00822 | 0.30832 | 0.48387 |
| **FA** | rs76122535 | 2 | 2.04E+08 | G | C | 0.1344 | 0.3609 | 0.0619 | 34.0142 | 5.57E-09 | -0.04870 | 0.01134 | 0.13342 | 0.00002 |
| **MD** | rs112431991 | 17 | 4.40E+07 | G | A | 0.2224 | 0.2871 | 0.0517 | 30.8625 | 2.81E-08 | 0.03569 | 0.01102 | 0.20849 | 0.00120 |
| **MD** | rs11813268 | 10 | 1.06E+08 | T | C | 0.1534 | 0.3459 | 0.0598 | 33.4827 | 7.31E-09 | -0.02584 | 0.01061 | 0.15591 | 0.01492 |
| **MD** | rs33599 | 5 | 8.28E+07 | A | G | 0.2633 | -0.3129 | 0.0490 | 40.7279 | 1.79E-10 | 0.00808 | 0.00844 | 0.27238 | 0.33841 |
| **MD** | rs4150221 | 5 | 1.40E+08 | C | T | 0.2634 | -0.2661 | 0.0486 | 29.9891 | 4.40E-08 | 0.00136 | 0.00854 | 0.26595 | 0.87396 |
| **MD** | rs67827860 | 5 | 8.29E+07 | T | C | 0.1917 | 0.6667 | 0.0546 | 148.9946 | 3.96E-34 | 0.00522 | 0.00949 | 0.19765 | 0.58251 |
| **WMH** | rs12571024 | 10 | 1.06E+08 | T | C | 0.4707 | -0.0592 | 0.0101 | 34.2425 | 4.96E-09 | 0.01527 | 0.00807 | 0.43301 | 0.05850 |
| **WMH** | rs12615761 | 2 | 4.31E+07 | G | T | 0.1801 | -0.0728 | 0.0126 | 33.4793 | 7.32E-09 | 0.00542 | 0.00983 | 0.18324 | 0.58169 |
| **WMH** | rs12921170 | 16 | 8.72E+07 | G | A | 0.4182 | -0.0583 | 0.0097 | 35.8990 | 2.11E-09 | -0.00110 | 0.00788 | 0.42913 | 0.88935 |
| **WMH** | rs2293767 | 7 | 1.00E+08 | A | G | 0.2894 | -0.0592 | 0.0106 | 31.0728 | 2.52E-08 | 0.00794 | 0.00828 | 0.29203 | 0.33760 |
| **WMH** | rs275350 | 6 | 1.51E+08 | C | G | 0.4135 | 0.0590 | 0.0098 | 36.4719 | 1.58E-09 | 0.00516 | 0.00770 | 0.40448 | 0.50272 |
| **WMH** | rs34974290 | 17 | 7.39E+07 | A | G | 0.1861 | 0.1096 | 0.0122 | 80.0315 | 4.03E-19 | 0.01638 | 0.01006 | 0.17787 | 0.10346 |
| **WMH** | rs4525538 | 17 | 4.31E+07 | A | G | 0.3726 | -0.0726 | 0.0100 | 53.0785 | 3.32E-13 | 0.02306 | 0.00792 | 0.37410 | 0.00357 |
| **WMH** | rs7596872 | 2 | 5.61E+07 | A | C | 0.1015 | 0.1222 | 0.0160 | 58.3170 | 2.34E-14 | 0.01810 | 0.01315 | 0.09996 | 0.16869 |

Abbreviations: FA, fractional anisotropy; MD, mean diffusivity; WMH, white matter hyperintensities; Chr, chromosome; SE, standard error; Pos, position; EAF, effect allele frequency; SNP, single nucleotide polymorphism

**Supplement table 3.** Results for MR-Egger (intercept), MR-PRESSO, Cochran's Q test of migraine for WMLs

| **Risk factors** | **Outcome** | **MR-Egger Test** | | | **PRESSO Test** | | **Cochran’s Q Test** | | |
| --- | --- | --- | --- | --- | --- | --- | --- | --- | --- |
|  |  | **Intercept** | **SE** | **P-value** | **P-value** | **P-Outlier-corrected** | **Q** | **Q-df** | **P-value** |
| Migraine | FA | 0.008 | 0.024 | 0.731 | 0.389 | NA | 36.911 | 35 | 0.38 |
| Migraine | MD | -0.018 | 0.024 | 0.458 | 0.571 | NA | 33.908 | 35 | 0.52 |
| Migraine | WMH | -0.001 | 0.008 | 0.852 | 0.217 | 0.562 | 9.841 | 35 | 0.21^a^ |

Abbreviations: FA, fractional anisotropy; MD, mean diffusivity; WMH, white matter hyperintensities; MR-Egger: Mendelian randomization-Egger; SE: standard error.

a, use multiplicative random effects

**Supplement table 4.** Results for MR-Egger (intercept), MR-PRESSO, Cochran's Q test of WMLs for migraine

| **Risk factors** | **Outcome** | **MR-Egger Test** | | | **PRESSO Test** | | **Cochran’s Q Test** | | |
| --- | --- | --- | --- | --- | --- | --- | --- | --- | --- |
|  |  | **Intercept** | **SE** | **P-value** | **P-value** | **P-Outlier-corrected** | **Q** | **Q-df** | **P-value** |
| FA | Migraine | 0.009 | 0.022 | 0.705 | 0.988 | 0.980 | 19.344 | 7 | 0.454^a^ |
| MD | Migraine | -0.002 | 0.028 | 0.941 | 0.998 | 0.981 | 17.658 | 4 | 0.987^a^ |
| WMH | Migraine | -0.028 | 0.016 | 0.136 | 0.707 | 0.681 | 17.914 | 7 | 0.696^a^ |

Abbreviations: FA, fractional anisotropy; MD, mean diffusivity; WMH, white matter hyperintensities; MR-Egger: Mendelian randomization-Egger; SE: standard error.

a, use multiplicative random effects

Supplement figure 1: Scatter plots of the causal effects of migraine with risk for FA


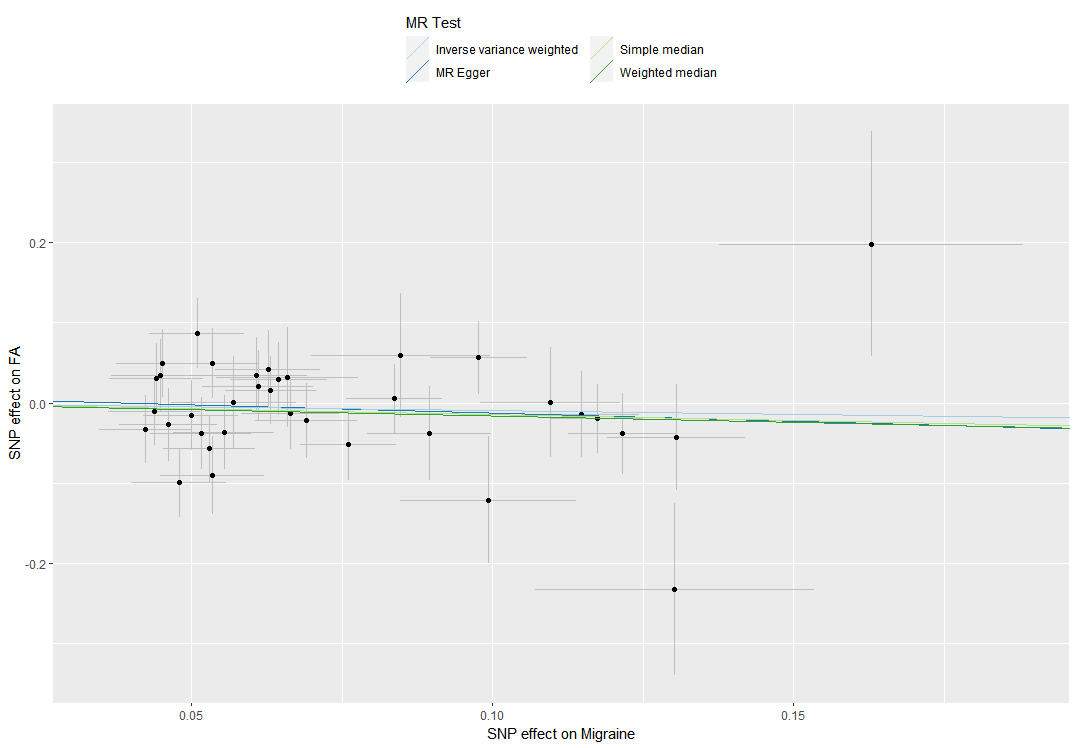


Supplement figure 2: Scatter plots of the causal effects of migraine with risk for MD


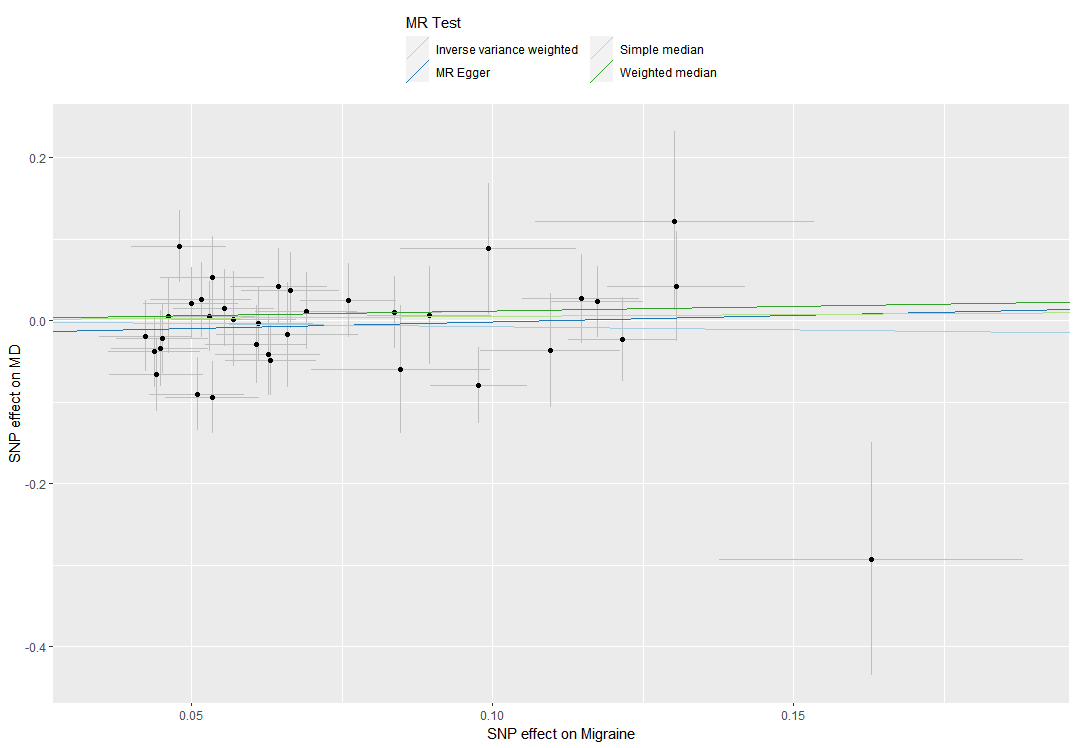


Supplement figure 3: Scatter plots of the causal effects of migraine with risk for WMH


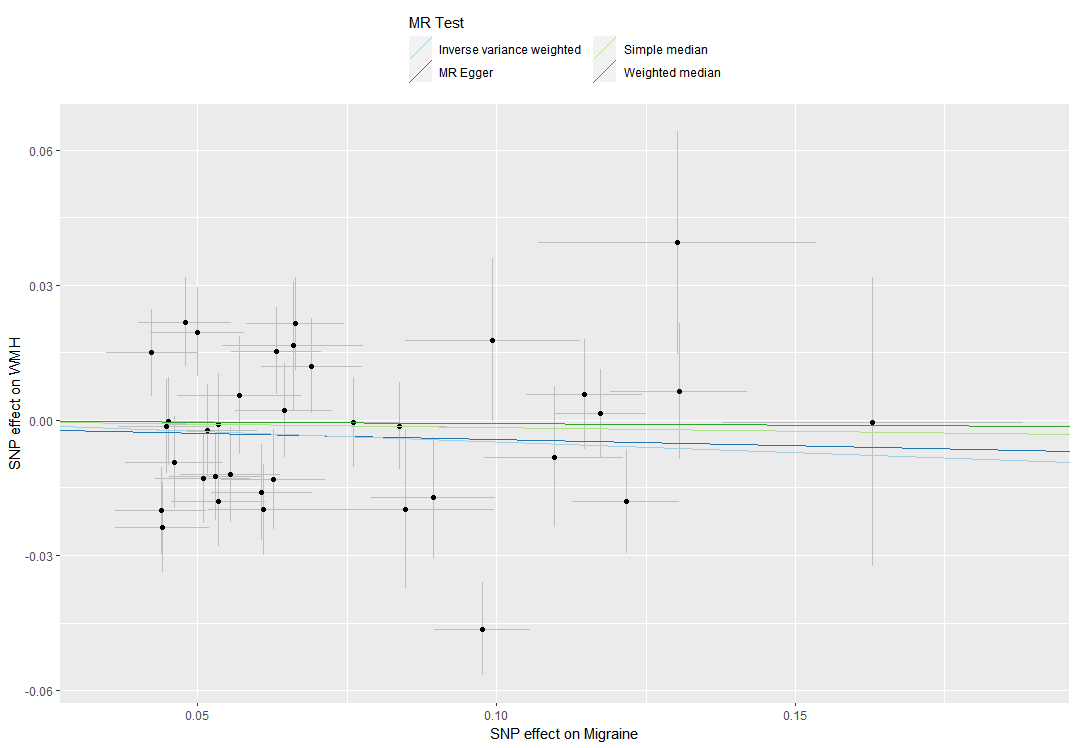


Supplement figure 4: Forest plots of the causal effects between migraine-related SNPs and FA


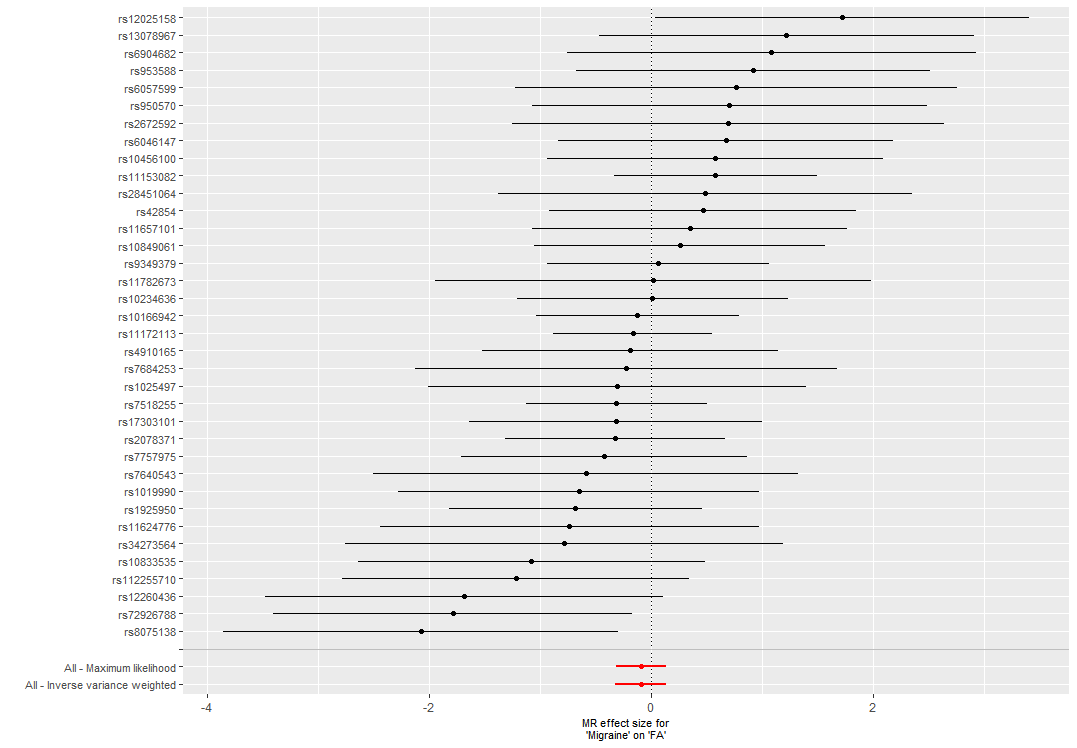


Supplement figure 5: Forest plots of the causal effects between migraine-related SNPs and MD


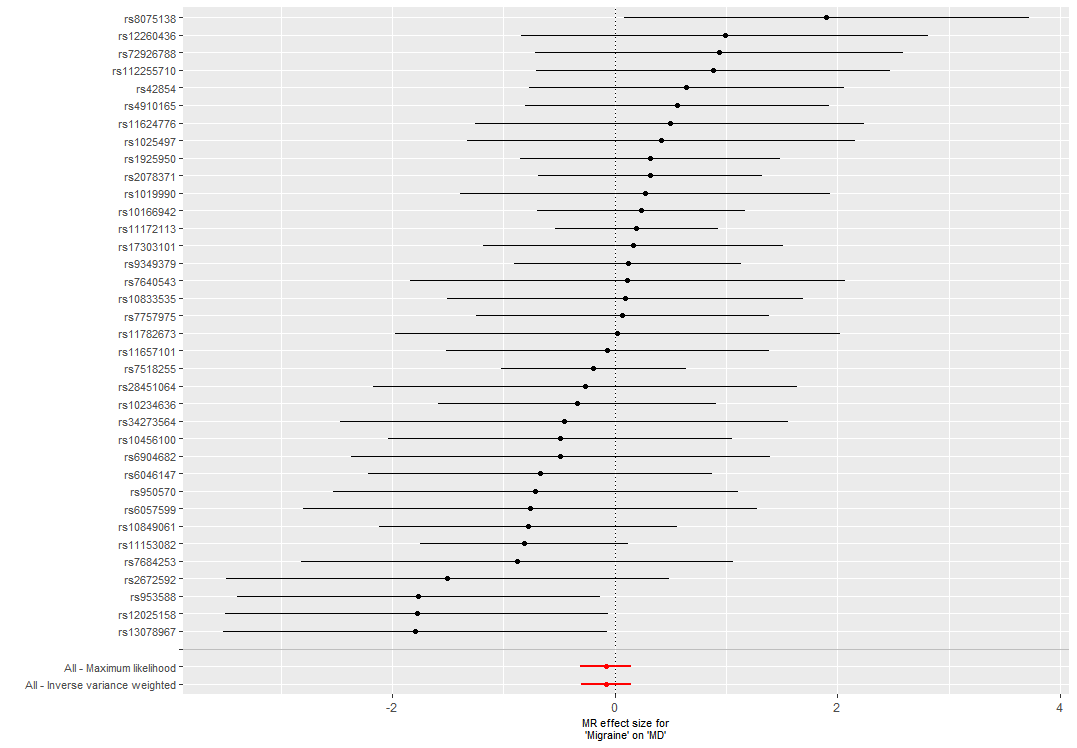
 Supplement figure 6: Forest plots of the causal effects between migraine-related SNPs and WMH


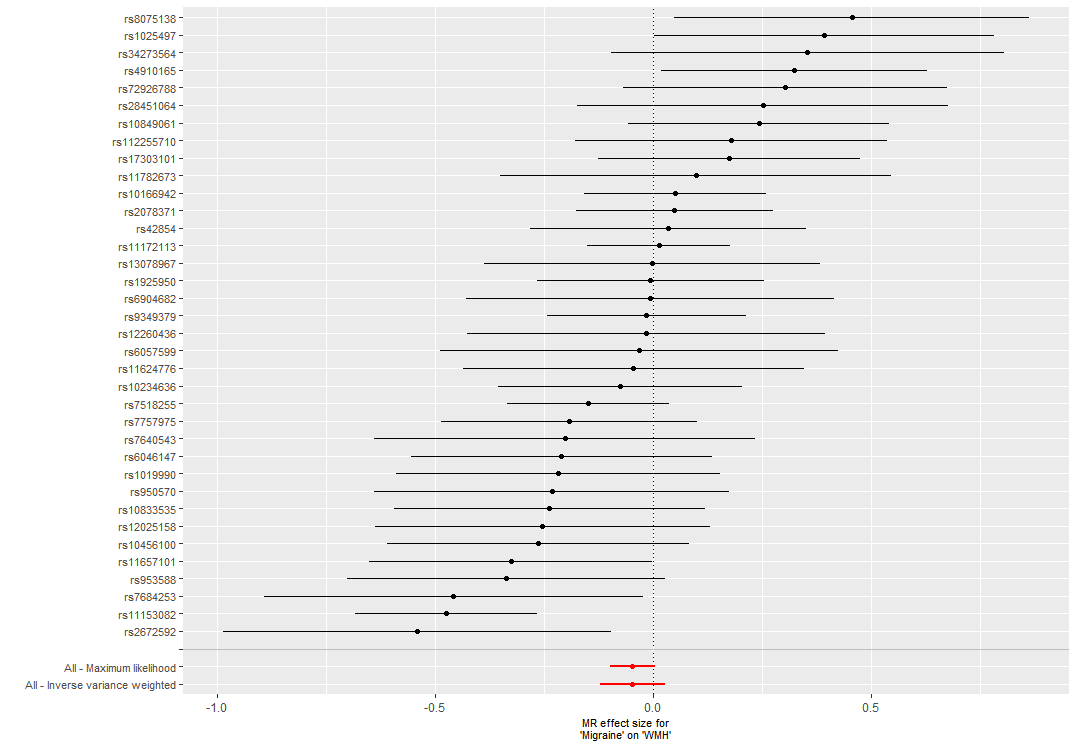


Supplement figure 7: Leave-one-out sensitivity analysis for migraine on FA


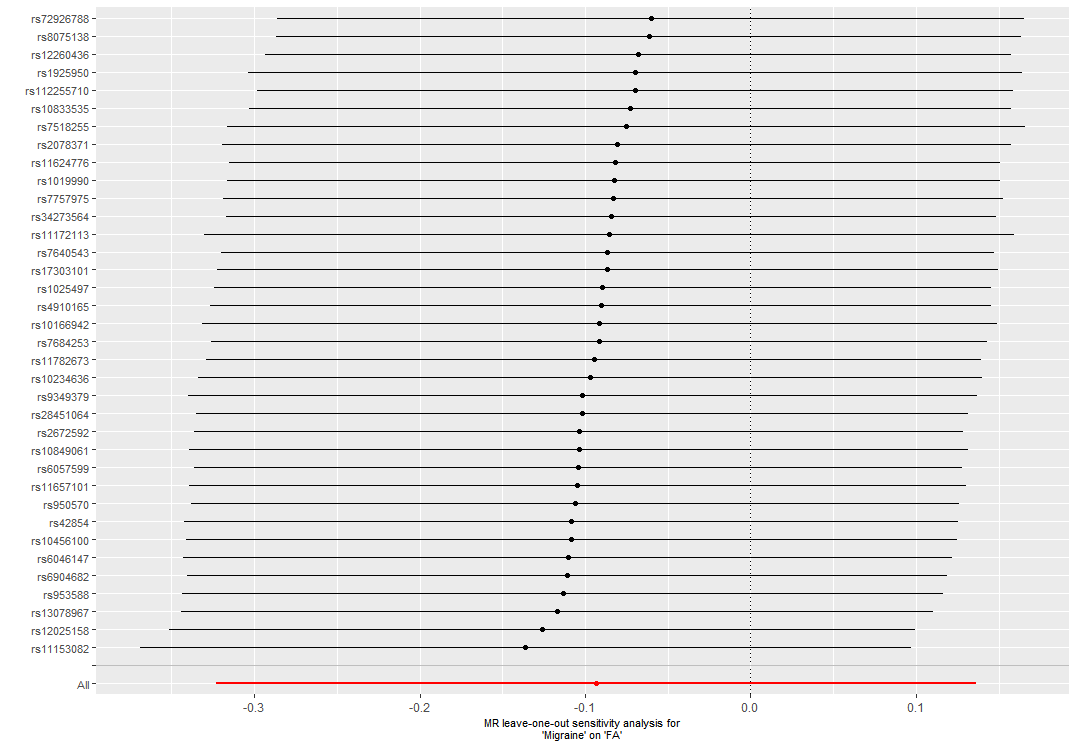


Supplement figure 8: Leave-one-out sensitivity analysis for migraine on MD


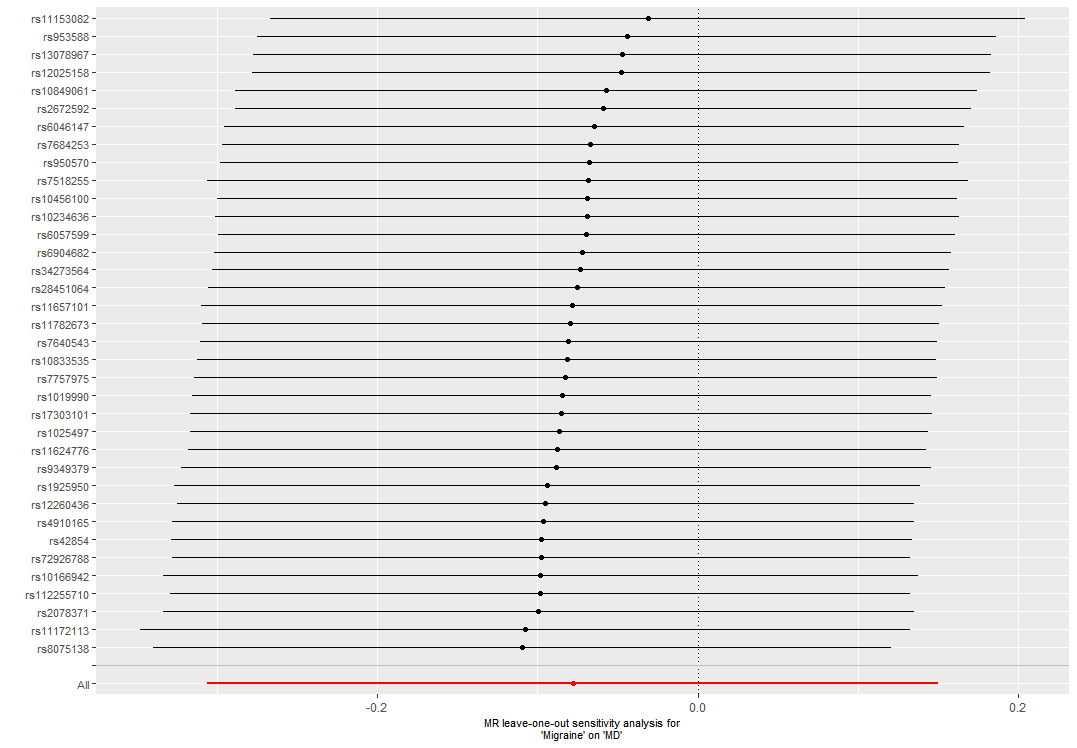


Supplement figure 9: Leave-one-out sensitivity analysis for migraine on WMH


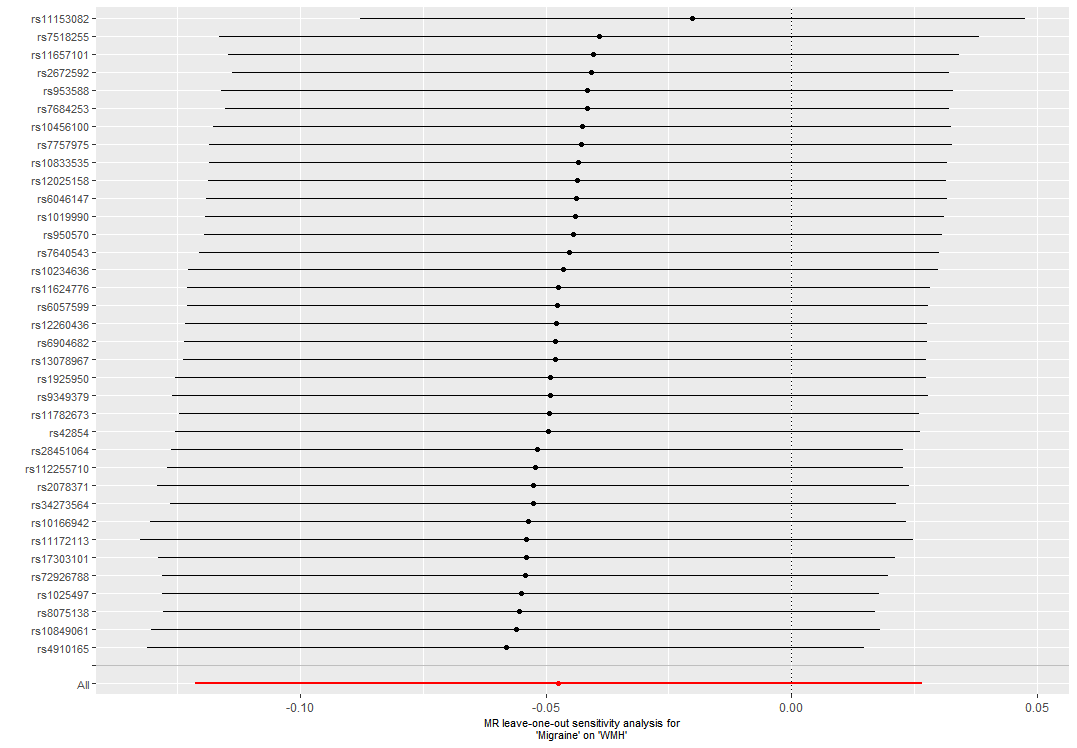


Supplement figure10: Scatter plots of the causal effects of FA with risk for migraine


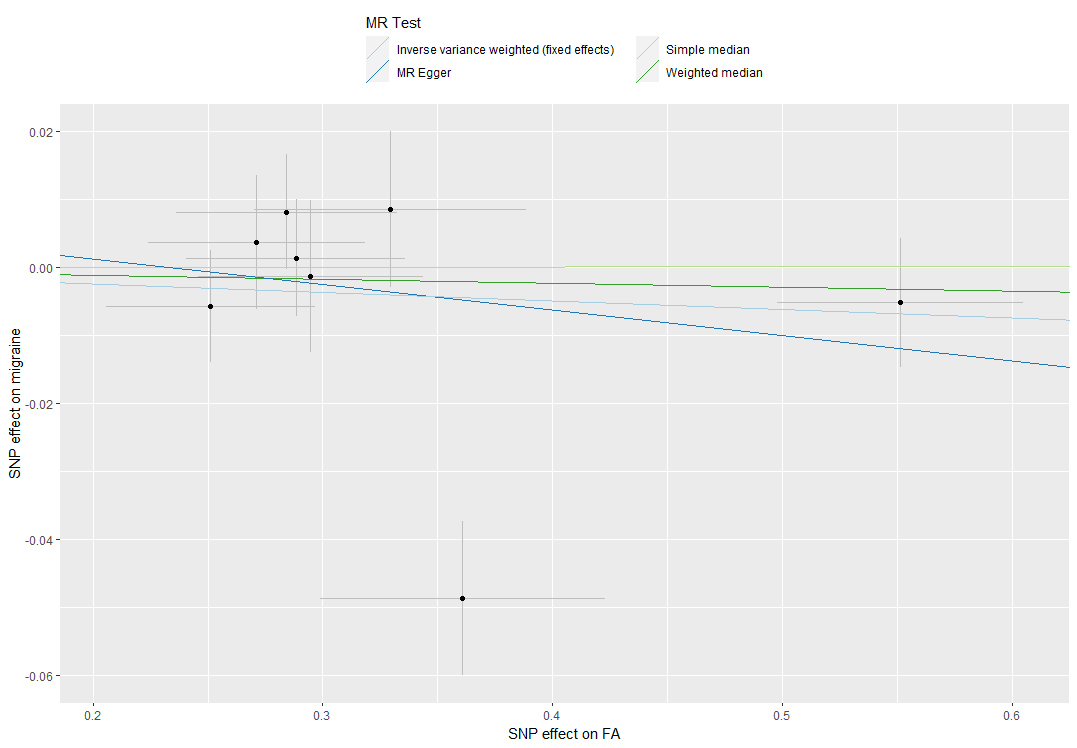


Supplement figure11: Scatter plots of the causal effects of MD with risk for migraine


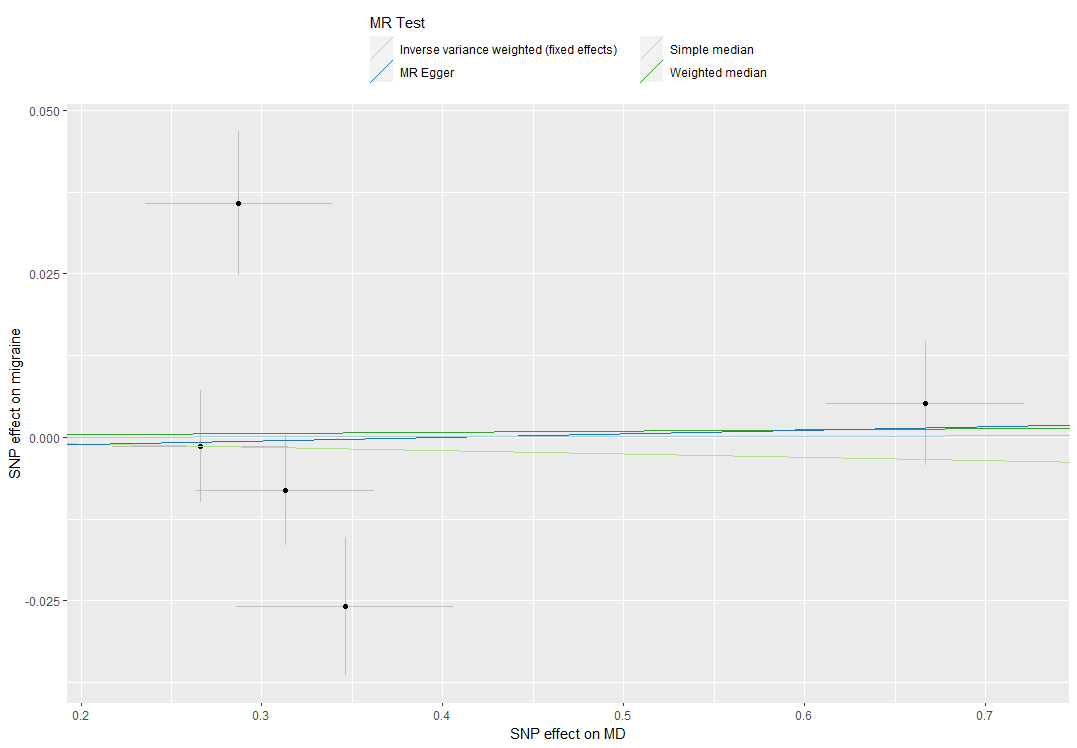


Supplement figure12: Scatter plots of the causal effects of WMH with risk for migraine


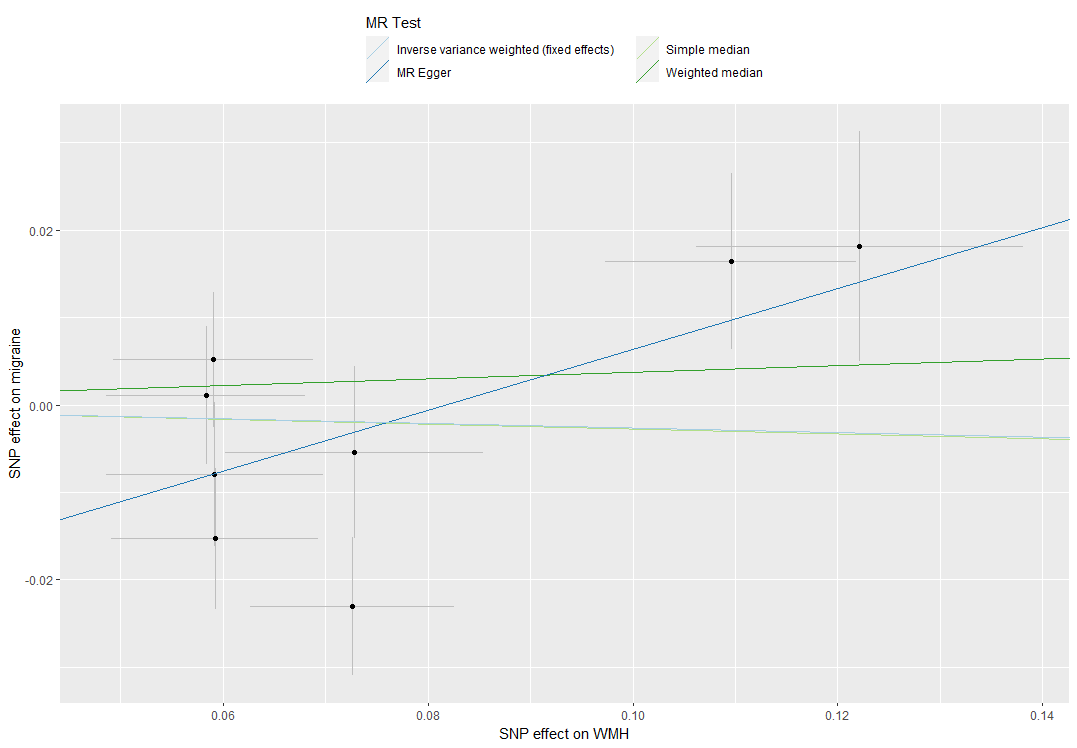


Supplement figure 13: Forest plots of the causal effects between FA SNPs and migraine


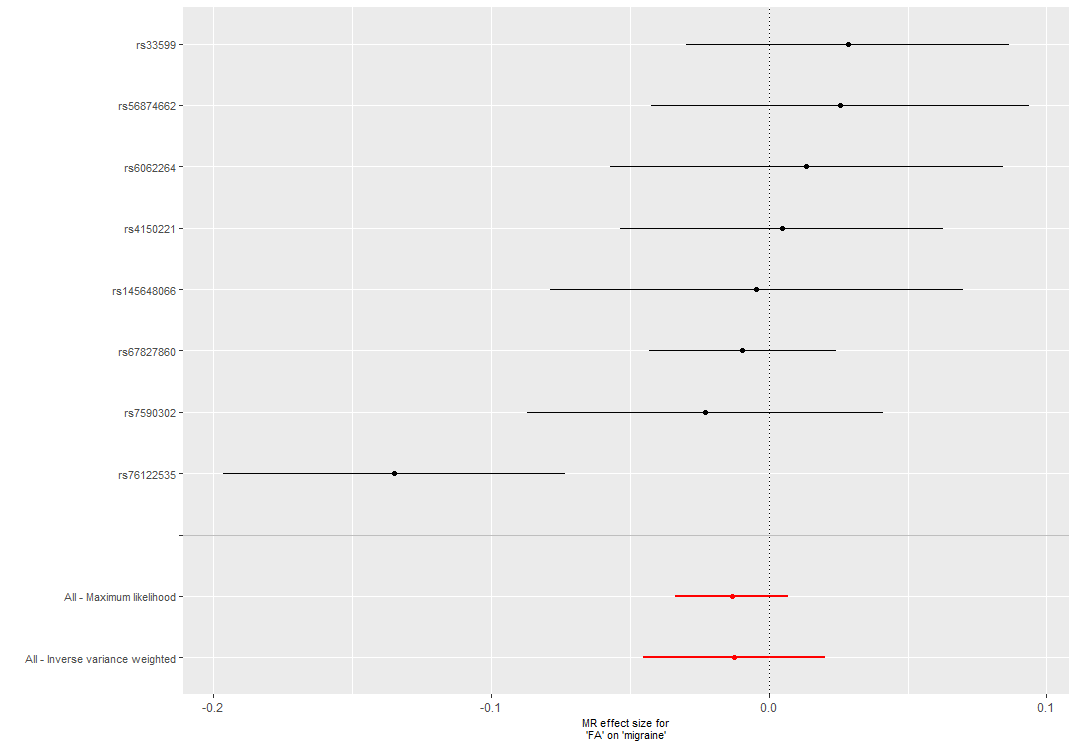


Supplement figure 14: Forest plots of the causal effects between MD SNPs and migraine


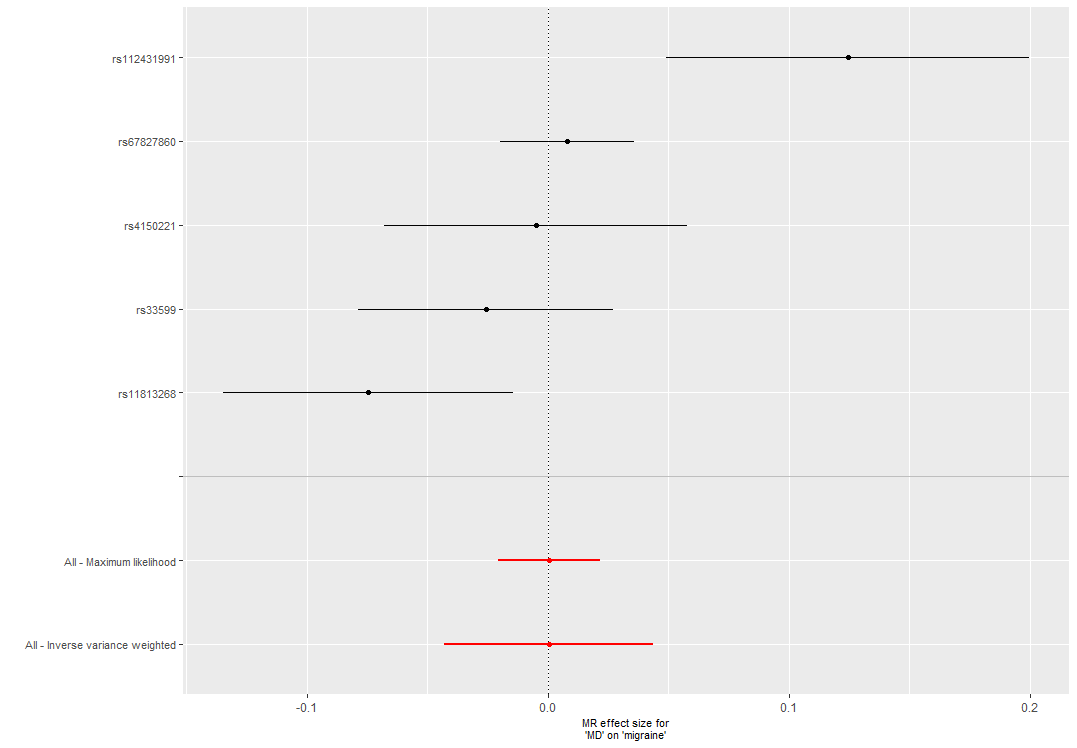


Supplement figure 15: Forest plots of the causal effects between WMH SNPs and migraine


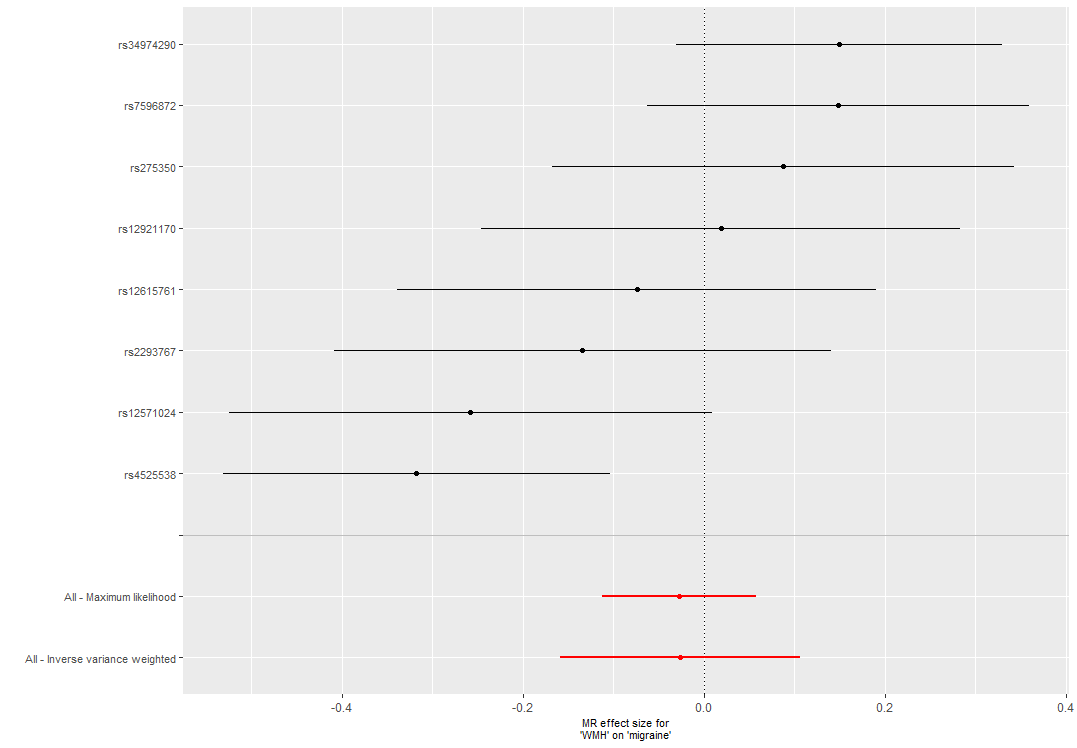
 Supplement figure16: Leave-one-out sensitivity analysis for FA on migraine


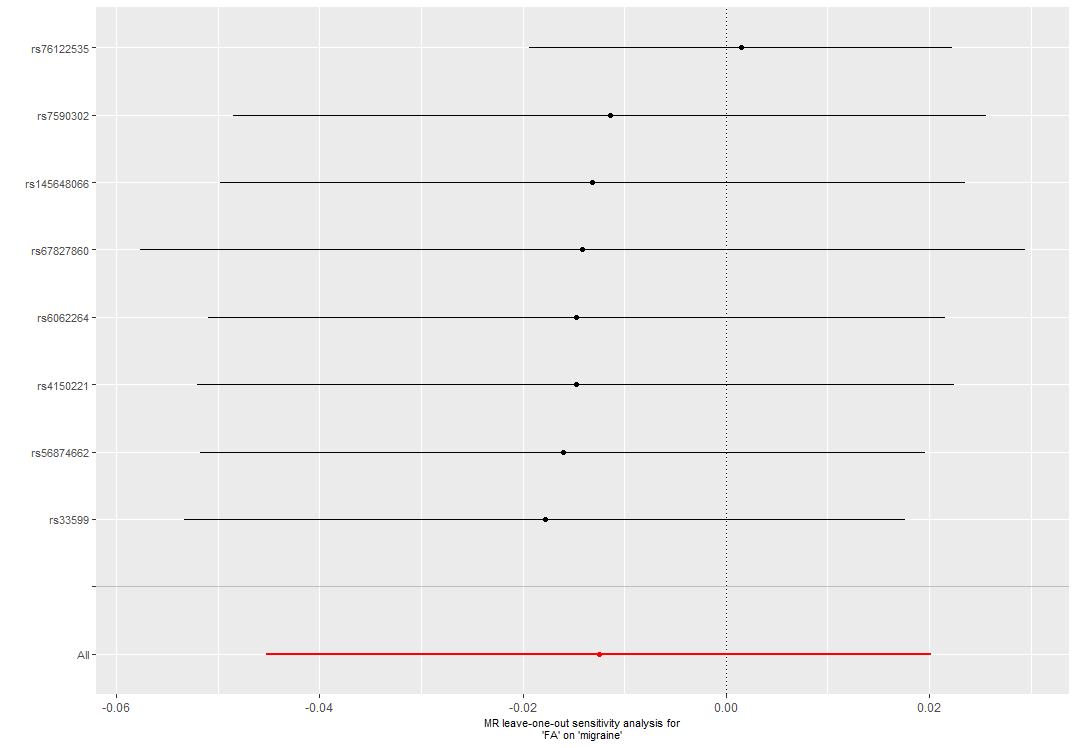


Supplement figure17: Leave-one-out sensitivity analysis for MD on migraine


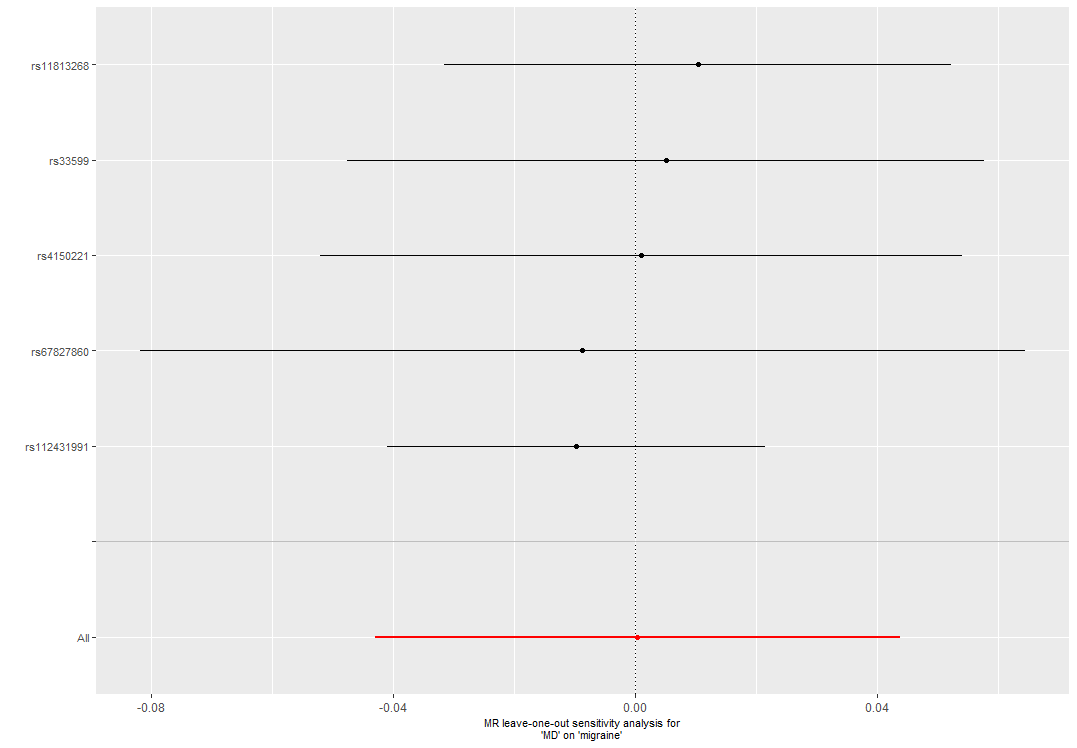


Supplement figure18: Leave-one-out sensitivity analysis for WMH on migraine


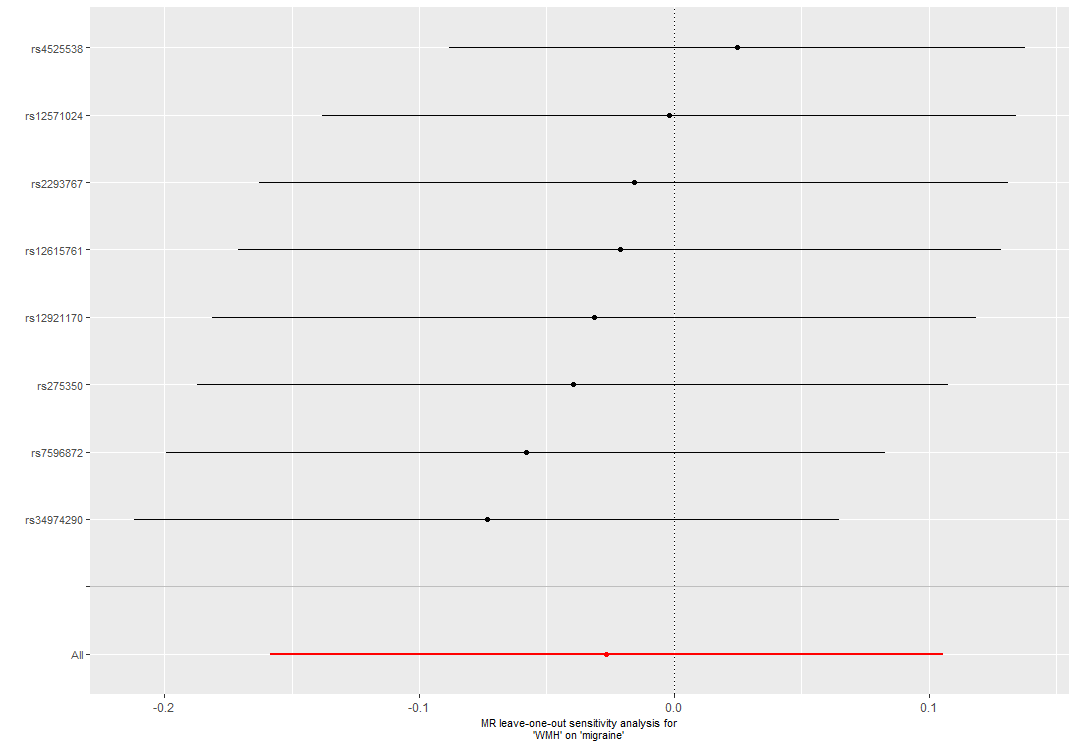

Supplement: Supplementary file 1 — Supplementary Information 1. [file 41598_2023_38182_MOESM1_ESM.docx]
