# Supplementary material for: Migraine and white matter lesions: a mendelian randomization study
Source: Sci Rep. 2023 Jul 6;13:10984. doi: 10.1038/s41598-023-38182-x (PMC10326014; doi:10.1038/s41598-023-38182-x)
Supplement: Supplementary file 2 — Supplementary Information 2. [file 41598_2023_38182_MOESM2_ESM.docx]

**International Headache Genetics Consortium Members**

Verneri Anttila^1,2,3^, Ville Artto^4^, Andrea C Belin^5^, Anna Bjornsdottir^6^, Gyda Bjornsdottir^7^, Dorret I Boomsma^8^, Sigrid Børte^9,10,11^, Mona A Chalmer^12^, Daniel I Chasman^13,14^, Bru Cormand^15^, Ester Cuenca-Leon^16^, George Davey-Smith^17^, Irene de Boer^18^, Martin Dichgans^19,20^, Tonu Esko^21^, Tobias Freilinger^22,23^, Padhraig Gormley^24^, Lyn R Griffiths^25^, Eija Hämäläinen^26^, Thomas F Hansen^12,27^, Aster VE Harder^18,28^, Heidi Hautakangas^26^, Marjo Hiekkala^29^, Maria G Hrafnsdottir^30^, M. Arfan Ikram^31^, Marjo-Riitta Järvelin^32,33,34,35^, Risto Kajanne^26^, Mikko Kallela^4^, Jaakko Kaprio^26^, Mari Kaunisto^29^, Lisette JA Kogelman^12^, Espen S Kristoffersen^36,37,38^, Christian Kubisch^39^, Mitja Kurki^40^, Tobias Kurth^41^, Lenore Launer^42^, Terho Lehtimäki^43^, Davor Lessel^39^, Lannie Ligthart^8^, Sigurdur H Magnusson^7^, Rainer Malik^19^, Bertram Müller-Myhsok^44^, Carrie Northover^45^, Dale R Nyholt^46^, Jes Olesen^12^, Aarno Palotie^26,47^, Priit Palta^26^, Linda M Pedersen^48^, Nancy Pedersen^49^, Matti Pirinen^26,50,51^, Danielle Posthuma^52^, Patricia Pozo-Rosich^53^, Alice Pressman^54^, Olli Raitakari^55,56,57^, Caroline Ran^5^, Gudrun R Sigurdardottir^6^, Hreinn Stefansson^7^, Kari Stefansson^7^, Olafur A Sveinsson^30^, Gisela M Terwindt^18^, Thorgeir E Thorgeirsson^7^, Arn MJM van den Maagdenberg^18,28^, Cornelia van Duijn^58^, Maija Wessman^29,26^, Bendik S Winsvold^48,9,59^, John-Anker Zwart^48,9,10^

**International Headache Genetics Consortium Affiliations**

^1^Analytical and Translational Genetics Unit, Department of Medicine, Massachusetts General Hospital and Harvard Medical School, Boston, Massachusetts, USA, ^2^Program in Medical and Population Genetics, Broad Institute of MIT and Harvard, Cambridge, Massachusetts, USA, ^3^Stanley Center for Psychiatric Research, Broad Institute of MIT and Harvard, Cambridge, Massachusetts, USA, ^4^Department of Neurology, Helsinki University Central Hospital, Helsinki, Finland, ^5^Department of Neuroscience, Karolinska Institutet, Stockholm, Sweden, ^6^Neurology private practice, Laeknasetrid, Reykjavik, Iceland, ^7^deCODE genetics/Amgen Inc., Reykjavik, Iceland, ^8^Netherlands Twin Register, Department of Biological Psychology, Vrije Universiteit, Amsterdam, the Netherlands, ^9^K.G. Jebsen Center for Genetic Epidemiology, Department of Public Health and Nursing, Faculty of Medicine and Health Sciences, Norwegian University of Science and Technology, Trondheim, Norway, ^10^Institute of Clinical Medicine, Faculty of Medicine, University of Oslo, Oslo, Norway, ^11^Research and Communication Unit for Musculoskeletal Health, Department of Research, Innovation and Education, Division of Clinical Neuroscience, Oslo University Hospital, Oslo, Norway, ^12^Danish Headache Center, Department of Neurology, Copenhagen University Hospital, Copenhagen, Denmark, ^13^Department of Medicine, Division of Preventive Medicine, Brigham and Women's Hospital, Boston, Massachusetts, USA, ^14^Harvard Medical School, Boston, Massachusetts, USA, ^15^Department of Genetics, Spain Centre for Biomedical Network Research on Rare Diseases, University of Barcelona, Barcelona, Spain, ^16^Pediatric Neurology Research Group, Vall d'Hebron Research Institute, Barcelona, Spain, ^17^University of Bristol/Medical Research Council Integrative Epidemiology Unit, University of Bristol, Bristol, UK, ^18^Department of Neurology, Leiden University Medical Centre, Leiden, the Netherlands, ^19^Institute for Stroke and Dementia Research, University Hospital, LMU Munich, Munich, Germany, ^20^Munich Cluster for Systems Neurology, Munich, Germany, ^21^Estonian Biobank Registry, the Estonian Genome Center, University of Tartu, Tartu, Estonia, ^22^Department of Neurology, Klinikum Passau, Passau, Germany, ^23^Department of Neurology and Epileptology, Hertie Institute for Clinical Brain Research, University of Tuebingen, Tuebingen, Germany, ^24^GSK Inc., Cambridge, Massachusetts, USA, ^25^Centre for Genomics and Personalised Health, Queensland University of Technology, Brisbane, Queensland, Australia, ^26^Institute for Molecular Medicine Finland, Helsinki Institute of Life Science, University of Helsinki, Helsinki, Finland, ^27^Novo Nordic Foundation Center for Protein Research, Copenhagen University, Copenhagen, Denmark, ^28^Department of Human Genetics, Leiden University Medical Centre, Leiden, the Netherlands, ^29^Folkhälsan Research Center, Helsinki, Finland, ^30^Landspitali University Hospital, Reykjavik, Iceland, ^31^Department of Epidemiology, Erasmus University Medical Center, Rotterdam, the Netherlands, ^32^Department of Epidemiology and Biostatistics, MRC-PHE Centre for Environment and Health, School of Public Health, Imperial College London, London, UK, ^33^Center for Life Course Health Research, Faculty of Medicine, University of Oulu, Oulu, Finland, ^34^Unit of Primary Health Care, Oulu University Hospital, OYS, Oulu, Finland, ^35^Department of Life Sciences, College of Health and Life Sciences, Brunel University London, London, UK, ^36^Research and Communication Unit for Musculoskeletal Health, Department of Research, Innovation and Education, Division of Clinical Neuroscience, Akershus University Hospital and University of Oslo, Oslo, Norway, ^37^Department of General Practice, Institute of Health and Society, University of Oslo, Oslo, Norway, ^38^Department of Neurology, Akershus University Hospital, Lørenskog, Norway, ^39^Institute of Human Genetics, University Medical Center Hamburg-Eppendorf, Hamburg, Germany, ^40^Psychiatric and Neurodevelopmental Genetics Unit, Department of Medicine, Massachusetts General Hospital, Boston, Massachusetts, USA, ^41^Institute of Public Health, Charité – Universitätsmedizin, Berlin, ^42^Laboratory of Epidemiology and Population Sciences, Intramural Research Program, National Institute on Aging, Bethesda, Maryland, USA, ^43^Department of Clinical Chemistry, Fimlab Laboratories, and Finnish Cardiovascular Research Center - Tampere, Faculty of Medicine and Health Technology, Tampere University, Tampere, Finland, ^44^Max Planck Institute of Psychiatry, Munich, Germany, ^45^23&Me Inc., Mountain View, California, USA, ^46^School of Biomedical Sciences, Faculty of Health, Centre for Genomics and Personalised Health, Centre for Data Science, Queensland University of Technology, Brisbane, Queensland, Australia, ^47^University of Helsinki, Helsinki, Finland, ^48^Department of Research, Innovation and Education, Division of Clinical Neuroscience, Oslo University Hospital, Oslo, Norway, ^49^Department of Medical Epidemiology and Biostatistics, Karolinska Institutet, Stockholm, Sweden, ^50^Department of Mathematics and Statistics, University of Helsinki, Helsinki, Finland, ^51^Department of Public Health, University of Helsinki, Helsinki, Finland, ^52^Department of Complex Trait Genetics, Center for Neurogenomics and Cognitive Research, Neuroscience Campus Amsterdam, VU University, Amsterdam, The Netherlands, ^53^Headache Unit, Neurology Department, Vall d'Hebron University Hospital, Barcelona, Spain, ^54^Sutter Health, Sacramento, California, USA, ^55^Centre for Population Health Research, University of Turku, Turku University Hospital, Turku, Finland, ^56^Research Centre of Applied and Preventive Cardiovascular Medicine, University of Turku, Turku, Finland, ^57^Department of Clinical Physiology and Nuclear Medicine, Turku University Hospital, Turku, Finland, ^58^Department of Epidemiology, Erasmus University Medical Centre, Rotterdam, the Netherlands, ^59^Department of Neurology, Oslo University Hospital, Oslo, Norway
